# Solving the grand challenge of phenotypic integration: allometry across scales

**DOI:** 10.1007/s10709-022-00158-6

**Published:** 2022-07-20

**Authors:** François Vasseur, Adrianus Johannes Westgeest, Denis Vile, Cyrille Violle

**Affiliations:** 1 CEFE, University Montpellier, CNRS, EPHE, IRD, Montpellier, France; 2 LEPSE, University Montpellier, INRAE, Institut Agro, Montpellier, France

**Keywords:** Allometry, Phenotypic integration, Genome size, Metabolic scaling, Trait relationships

## Abstract

Phenotypic integration is a concept related to the cascade of trait relationships from the lowest organizational levels, i.e. genes, to the highest, i.e. whole-organism traits. However, the cause-and-effect linkages between traits are notoriously difficult to determine. In particular, we still lack a mathematical framework to model the relationships involved in the integration of phenotypic traits. Here, we argue that allometric models developed in ecology offer testable mathematical equations of trait relationships across scales. We first show that allometric relationships are pervasive in biology at different organizational scales and in different taxa. We then present mechanistic models that explain the origin of allometric relationships. In addition, we emphasized that recent studies showed that natural variation does exist for allometric parameters, suggesting a role for genetic variability, selection and evolution. Consequently, we advocate that it is time to examine the genetic determinism of allometries, as well as to question in more detail the role of genome size in subsequent scaling relationships. More broadly, a possible—but so far neglected—solution to understand phenotypic integration is to examine allometric relationships at different organizational levels (cell, tissue, organ, organism) and in contrasted species.

## Building complex organisms: an allometric perspective

The development of large and complex multicellular organisms requires the coordination and integration of a multitude of processes at different organizational levels (Pigliucci 2007; Zinner et al. 2020). Phenotypic integration (i.e. how complex organisms are built and how phenotypic traits are connected and coordinated) has been examined from a wide variety of approaches and research fields, including physiology, evolutionary biology, ecology and allometry (see for instance Schlichting and Pigliucci 1995; Pigliucci et al. 1996; Klingenberg 1996; Murren 2002; Pigliucci 2003; Klingenberg and Marugan-Lobon 2013; Klingenberg 2014; Klingenberg 2016; Watanabe 2022). However, joint empirical efforts at the interface of those disciplines warrant further study. One reason is that the study of trade-offs and strategies in comparative ecology has largely occurred independently of the study of phenotypic integration in evolutionary biology. Indeed, comparative ecology has mainly focused on global trends and trait-trait relationships across species, taking advantage of large interspecific databases (Brosse et al. 2021; Kattge et al. 2011; Díaz et al. 2016; Mouillot et al. 2021; Toussaint et al. 2021); while evolutionary biology has mainly focused on investigating the mechanisms and consequences of genetic correlations within species (Steppan et al. 2002; Arnold et al. 2008; Penna et al. 2017; Milocco and Salazar-Ciudad 2022).

**Fig. 1:**
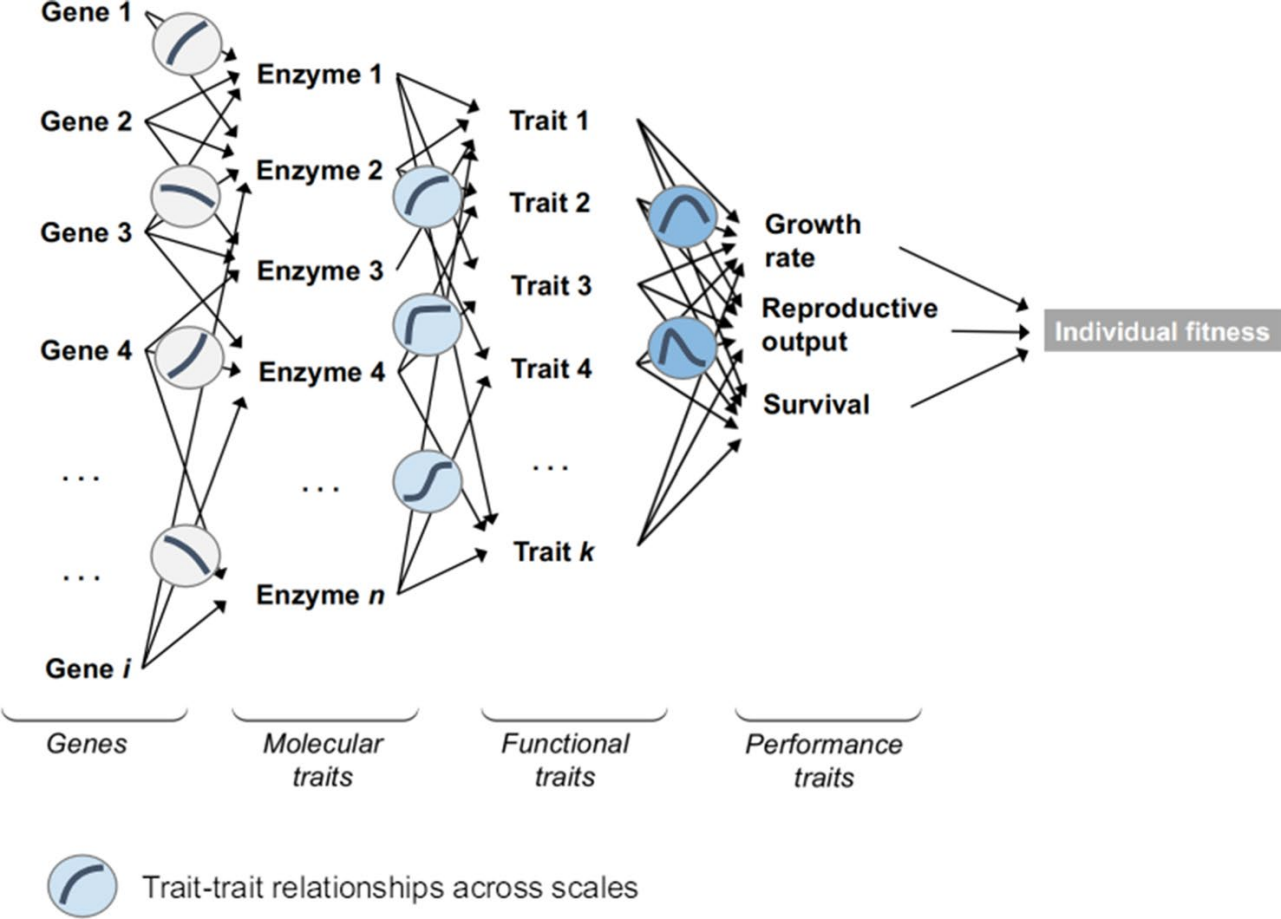
Arnold’s view of phenotypic integration. The pyramidal cascade of trait relationships from genes to organism’s fitness is represented. Here, the trait-trait relationships between two successive organizational levels are hypothetical

Following Arnold’s definition (Arnold 1983), phenotypic integration can be represented as a pyramid, in which traits related to gene products (RNA, enzymes) are at the bottom, and performance-related traits (growth rate, fecundity) at the top (Fig. 1). Ecological theory proposes a similar hierarchy of trait integration from genes to fitness (Violle et al. 2007). Basically, a trait Y is said to be integrated when it results from the effect of one or several traits X_i_ (considered at “lower degrees of phenotypic integration”). Traits at different organizational levels are assumed to be connected by scaling relationships or functions (Fig. 1). For instance, gene expression impacts RNA content, which in turn impacts cell metabolism and size, which in turn impacts organ physiology and size, and so on. Following Arnold’s view of phenotypic integration, a genetic variation impacting a trait should also impact the traits that are at higher levels and that result from the effect of the focal trait, with possible feedback effects between consecutive levels of integration. This is corroborated by recent findings, in which the rate of adaptive substitution (i.e. genetic variation that impacts organismal fitness) along the genome increases with the degree of phenotypic integration (Zhang 2018). However, the pyramidal view of phenotypic integration proposed by Arnold largely remains theoretical: the ‘cause-and-effect’ cascades, as well as the relationships involved, are notoriously difficult to establish, so is the hierarchy of phenotypic integration. Traits are generally organized into groups of interacting features, called modules, which are relatively independent from each others (e.g. floral traits versus vegetative traits in plants) (Wagner et al. 2007; Klingenberg 2008; Murren 2012; Diggle 2014). However, how modules and traits at different scales are connected to each other remains unclear.

One of the main consequences of a change in the size of an object is the modification of its surface area to volume ratio. This is explained by simple geometric considerations: the surface area, which allows exchanges of resources and energy between the internal medium and the environment, increases according to a square function; while the volume, a priori linked to the number of cells and thus to energy needs, increases according to a cubic function. As a result, the surface-to-volume ratio decreases with volume and mass, whatever the shape of the object (but see Bestova et al. 2021). In living things, these geometric constraints have profound consequences for resource use strategies. First, the rate of metabolic activity—measured through basal respiration rate—is expected to increase non-proportionally with organism size. Having a smaller surface-to-volume ratio, large individuals are expected to have a lower metabolic rate (Kleiber 1947) and to lose less heat per unit biomass (Bergmann’s rule) (Bergmann 1847; Blackburn et al. 1999). For example, an increase of only 10% in height and width of a cone representing a simplified tree would result in an increase of c. 20% in its surface area, and an increase of c. 33% in its volume. A direct consequence is that surface-related processes, such as carbon fixation and water loss, increase twice as fast as the increase in height and width. A tree with increased size will therefore be likely to use significantly more surface-related resources (e.g., transpiration of water, acquisition of carbon through photosynthesis). Moreover, volume-related processes, such as carbon consumption via cellular respiration, increase even more significantly (33%). This example is of course an unrealistic simplification (a tree is never as simple as a cone) of the physiological processes involved in complex organisms, but it illustrates the major, and asymmetric, influence of size variations on resource use, growth, and development (Ohlberger 2013; Malerba et al. 2017; Lindmark et al. 2019; Malerba and Marshall 2019).

**Fig. 2:**
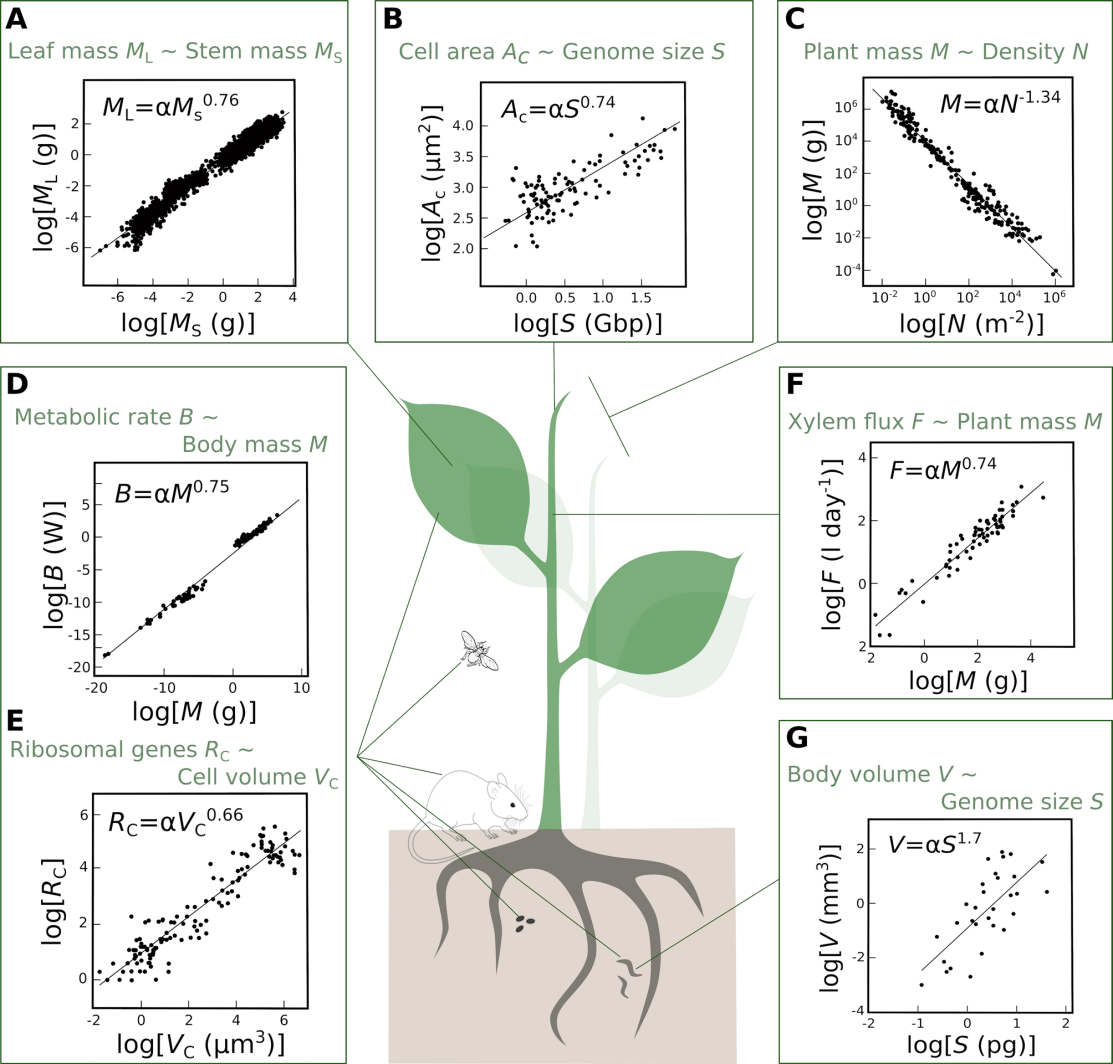
Examples of allometric relationships between scales. **A** Relationship between leaf mass (*M*_L_) and stem mass (*M*_S_) (Price et al. 2010). **B** Relationship between cell area (*A*_C_) and genome size (*S*) (Beaulieu et al. 2008). **C** Relationship between plant mass (*M*) and plant density (*N*) (Enquist et al. 1998). **D** Relationship between organism’s basal metabolic rate (*B*) and body mass (*M*) (West et al. 2002). **E** Relationship between the number of 16S/18S ribosomal genes per cell (*R*_C_) and cell volume (*V*_C_) (Gonzalez-de-Salceda and Garcia-Pichel 2021). **F** Relationship between xylem flux (*F*) and plant mass (*M*) (Enquist and Niklas 2001). **G** Relationship between flatworm body volume (*V*) and genome size (*S*) (Gregory et al. 2000)

Considering that the size of an object is the main determinant of its internal properties is the central tenet of allometry research, which, in its broad definition, is the study of the relationships between the size of a biological entity (e.g., genome, cell, organ, organism, population) and its morphological, physiological and metabolic traits (Niklas 1994). Allometric equations aim at modelling trait-trait relationships across organizational scales. They typically take the form of power-law functions *Y* = α*X*^β^, where *X* is a size-related trait and *Y* a morphological or physiological trait (Niklas 1994). Strikingly, the same equations are used to scale up from genome size to cell size (Kozłowski et al. 2003b; Beaulieu et al. 2008; Šímová and Herben 2011; Gonzalez-de-Salceda and Garcia-Pichel 2021), cell size to organ size (Gregory et al. 2000; Tisné et al. 2008; John et al. 2013), organ size to organism size (Stahl 1965; Gould 1971; Stevenson et al. 1995; Lindstedt and Schaeffer 2002; Shingleton et al. 2007; Poorter et al. 2015), body size to energy consumption (Huxley 1932; Kleiber 1932, 1947; DeLong et al. 2010; Capellini et al. 2010), as well as from organism size to population density (Enquist et al. 1998; Malerba and Marshall 2019) (see Fig. 2 for examples of allometric relationships at different organizational levels). We advocate that a promising approach to understand phenotypic integration is to study scaling relationships, because modelling such relationships is built from a mathematical toolkit aimed at linking one level of integration to another.

## Integrating traits across scales: biological regularity versus genetic diversity

The allometric equations used to connect one phenotypic level to another are generally linearized by log-transformation, and β becomes the slope of the relationships log(*Y*) = log(*α*) + βlog(*M*) (Fig. 2). Simple geometric models predict β = 2/3 because β must be determined by the ratios between surface area (square function) and volume (cubic function) (Rubner 1883; White and Seymour 2003). However, in 1932, Kleiber observed that energy consumption in animals—which spanned several orders of magnitude in size—scales with body mass with an allometric exponent β = ¾ (Kleiber 1932). This observation yielded many studies on the value that β actually takes for different traits in different taxa. Moreover, the question of a biological constant that applies to virtually all organisms has been the subject of intense debates in the scientific literature. Indeed, the sole existence of a biological constant would suggest that organisms are built under the same developmental constraints, which translates into universal scaling relationships. Moreover, if the same constants are observed at different levels of phenotypic integration (from genomes to whole organisms), this would have strong implications for our understanding, and the potential prediction, of biological structures and organization.

The introduction of fractal geometries into mechanistic models has revolutionized the field of allometry research. In the late 1990s and early 2000s, a series of papers laid the theoretical foundations for the Metabolic Scaling Theory (MST) (Enquist et al. 1999, 2007b; West et al. 1999a, b, 2001, 2002; Niklas and Enquist 2001; Brown et al. 2004; Price and Enquist 2007), which invokes the fractal-like geometry of vascular networks as the main determinant of several scaling relationships in both plants and animals. MST is primarily based on two central hypotheses: the network is space-filling and the branching is area-preserving, i.e. the sum of cross-sectional areas of daughter branches equals the cross-sectional area of the mother branch. These hypotheses led to predictions concerning the variation of organism morphology, the allocation of biomass and the physiological rates at the organism level (Fig. 2) (Savage et al. 2008). For instance, the organism’s basal metabolic rate is expected to scale with β = ¾ to the organism’s body mass due to the optimization of the internal transport distance for resources and energy (West et al. 1999b). In plants, the metabolic rate is expected to scale isometrically (i.e. with exponent β = 1, which directly translates strict proportionality) with total leaf area and total leaf biomass, and allometrically (with exponent β = ¾) with the mass of the plant (Enquist et al. 1998; West et al. 1999a; Price et al. 2007). These scaling expectations represent the master equations of the MST, from which other scaling relationships at different levels of phenotypic integration have been derived, such as metabolic activity, relative growth rate, xylem fluxes and net photosynthetic rate in plants (Enquist 2002; Price and Enquist 2007; Enquist et al. 2007b; Savage et al. 2008). MST predictions even extend to population dynamics and demography, as illustrated by the expected exponent of − 3/4 for the relationship between maximum plant density and plant size (Enquist et al. 1998). However, some of the mechanistic assumptions of MST remain controversial, in large part because the universality of the scaling exponent has been challenged theoretically and empirically (Dodds et al. 2001; Kozłowski and Konarzewski 2004; Glazier 2005; Coomes 2006; Russo et al. 2007).

The theoretical underpinnings of MST are based on the idea that natural selection has optimized vascular networks in order to minimize the energy required for resource distribution, which would shape quasi-universal allometric relationships within and between species (West et al. 1999b). Interspecific comparisons have revealed global patterns of covariation that generally confirm the predictions of MST, in both animals and plants (Fig. 2). Moreover, recent intraspecific studies in plants also demonstrated an allometric exponent of ¾ in wild tomatoes and in *A. thaliana* (Vasseur et al. 2012; Muir and Thomas-Huebner 2013, 2015). This suggests that allometric relationships result from strong constraints, which operate at different organizational levels independently from the taxonomic scale and which, on average, adhere to the predictions of MST. Yet, intraspecific variability in allometries are also frequently observed in animals (Kozłowski et al. 2003a; Sieg et al. 2009; Summers and Ord 2021), and in plants (Vasseur et al. 2012; Muir and Thomas-Huebner 2013, 2015). For instance, the rate of respiratory metabolism has been found to scale isometrically (*i.e*. with exponent β ≈ 1) with plant biomass in herbaceous plants and tree seedlings (Reich et al. 2006; Cheng et al. 2010). Other studies in plants (Enquist et al. 2007a; Mori et al. 2010) and mammals (Kolokotrones et al. 2010), showed that respiration rate scales differently for organisms with small body mass and large body mass. In plants, competition for light results in significant variations in allometric relationships in trees (Russo et al. 2007; Lines et al. 2012). Strikingly, the studies reporting that intraspecific plant allometries follow the ¾ rule on average, also showed that the allometric exponent can vary significantly around ¾ because of evolutionary adaptation to stressful environments (Vasseur et al. 2018b). Studies in plants also indicated that variation in allometric exponents are genetically associated with different carbon and water-use strategies (Vasseur et al. 2012, 2014; Muir and Thomas-Huebner 2015), as well as reproductive output and resistance to stress (Muir and Thomas-Huebner 2013; Vasseur et al. 2018b). Together, these recent findings support the idea that scaling relationships vary between populations as a consequence of evolutionary adaptation to different environments, but they generally center around ¾. Overall, it suggests that phenotypic integration obeys fundamental laws that are translated into the relative constancy of allometric relationships at different organizational levels. However, a key question related to the integration of phenotypic traits is how adaptation to contrasting environments is the result of, or is dependent on, substantial variations in allometric relationships (Pigliucci et al. 1996; Pélabon et al. 2014).

## Current prospects in allometry: toward a genetic synthesis?

Exploring the genetic determinism of intraspecific allometries represents a promising avenue to better understand how organisms are built. Recent efforts have been devoted to the identification and characterization of genetic diversity in allometric relationships (Long et al. 2006; Qin et al. 2012, 2013; Vasseur et al. 2012, 2018b; Muir and Thomas-Huebner 2015). These works suggest that selection must operate on the genes that impact allometric parameters such as β, because the same biomechanical constraints (notably those related to surface area-to-volume ratio) are expected to operate at different scales. Identifying those genes should be a research priority to determine which molecular pathways are involved in the integration of phenotypic traits with size. In addition, the genetic architecture of intraspecific variability in allometry is directly informative about how organisms are expected to evolve. The predominance of few pleiotropic genes with strong effects on allometries, for instance genes related to life history, would suggest evolution by abrupt increments and sharp variations (Paaby and Rockman 2013). By contrast, if allometries are determined by a multitude of genes along the genomes, each having only a small effect, then a more gradual and continuous evolution of organisms is expected, which is directly related to the integration of multiple traits in complex organisms, and which has been called the “cost of complexity” (Orr 2000; Wagner et al. 2008). Genome-wide association studies (GWAS) might help to clarify the oligo- and/or the polygenic nature of allometric variations by the screening of plant and animal populations at different organizational levels. Furthermore, the recent rise of next-generation sequencing technologies allows extensive genomic analyses of the identified genes. The genetic survey of natural populations through space and time can reveal genomic signatures of selection and clarify the demographic history of the investigated populations (Weigel 2012). For instance, comparing genetic and phenotypic differentiation between populations through Q_ST_/F_ST_ approaches (Leinonen et al. 2013) allows testing whether allometric parameters such as β vary between populations due to neutral differentiation such as genetic drift, or because of directional or balancing selection. This kind of approach coupling allometry with quantitative genetics and genomics has recently been applied to the model plant *Arabidopsis thaliana*. It first identified two major pleiotropic genes as responsible for allometric variation between plant growth rate and plant dry mass (Vasseur et al. 2012), suggesting that plants evolve through strong allometric increments. However, this study was performed on a population of recombinant inbred lines (RIL) artificially created by crossing two contrasted ecotypes. A similar study performed six years later on a collection of 450 ecotypes (Vasseur et al. 2018b) revealed by contrast that plant allometry has a more polygenic architecture, with many contributing genes and with the major gene identified affecting only around 1% of the phenotypic variance. Taking advantage of the extensive genetic information available for *A. thaliana*, this study also showed that genes related to allometric variations exhibited signatures of selection and they were clustered geographically along a latitudinal gradient. Despite these encouraging findings, our knowledge of the genetic determinism of organism allometries remains limited. Yet, genetic studies of allometric variations could shed light on the complex genotype to-phenotype relationships involved in the coordination of complex phenotypes. A more systematic analysis of intraspecific relationships at different levels of integration (genome, cell, organ, and whole organism) is needed, in different species and different environmental conditions. Moreover, testing the effect of natural or artificial selection in real time with experimental evolution is a promising avenue to examine the evolution of allometry and its role for organism adaptation to contrasted environments.

## Allometric scaling from genome size to whole organisms: the grand challenge

An alternative approach to explore phenotypic integration through the lens of allometry is to link scaling relationships at different organizational levels (i.e. from genome to cell, organ and whole organism). Each level has its own set of allometric expectations, with its own theoretical corpus. For instance, there are allometric expectations for the allometry of genome size versus cell size (Šímová and Herben 2011), and cell size versus organism size (Gregory et al. 2000), as well as organism size versus metabolic rate or growth rate (Brown et al. 2004; Savage et al. 2008). Connecting these expectations, made at different levels and from different biological considerations, around a unifying model represents the ultimate challenge in allometry research. However, and as said above, the questions surrounding the links of cause-and-effects between relationships and organizational levels are particularly difficult to address. If in theory we could build allometric relationships to directly connect the lower level of integration—genome size—to the upper—organism size and metabolism—, in practice allometric relationships are generally strong between two successive organizational levels—e.g., genome size versus cell size, organism size versus organ size—but they decrease in predictive power with increasing phenotypic scale (Knight and Beaulieu 2008).

We advocate here that a promising avenue to solve this issue relies on the measurement of genome size. Genome size is usually estimated by DNA content in pg, which is expected to vary a lot among species because of different sources of variability in genome composition (including the number of genes but above all the amount of non-coding DNA, transposable elements, structural variations, repeated sequences, etc.) (Schubert and Vu 2016). However, this measurement of genome size misses the key point: what really matters is not the total amount of DNA, but the number of different genes that are effectively expressed during organism growth and development, as well as the amount (and repetition) of DNA segments regulating gene transcription and translation (mostly ribosomal DNA). The amount of DNA and the level of gene expression might differ a lot due to different processes. First, large portions of the genome exhibit presence/absence polymorphisms between genotypes of the same species (Tan et al. 2012; Bush et al. 2013; González et al. 2013; Zhang et al. 2014; Jiang et al. 2015), which suggests strong variability in the number of expressed genes between these genotypes. To what extent this variability is related to phenotypic variation remains unknown so far (Weisweiler et al. 2019). Second, the level of heterozygosity could positively impact the number of expressed genes and/or alleles. This could partly explain the increase in organism vigour and size frequently reported in hybrids between homozygous lines (Charlesworth and Charlesworth 1999). Indeed, the number of heterozygous genes, as well as presence/absence alleles has been shown to be positively correlated to hybrid vigour in plants (Springer et al. 2009; Paschold et al. 2012; Hochholdinger and Baldauf 2018). The links between expressed genome size and plant vigour are also supported by the higher size or organs and whole organisms in polyploid lines (containing 4 N, 6 N, 8 N etc.) versus their diploid relatives (Chen 2010; Sattler et al. 2016). Consistent with allometric expectations, polyploids might exhibit larger organs and body size due to their enlarged genomes (Otto 2007; Lavania 2016). Accordingly, endopolyploidy at the cellular level is also associated with increased cell size in both plants and animals (Leitch and Dodsworth 2001; Neiman et al. 2017). However, polyploidy could also uncouple trait covariations and, thus, alter the pattern of phenotypic integration observed in diploids. This is notably the case for the traits related to leaf physiology and resource use (López-Jurado et al. 2022). Yet, to what extent polyploidy might break allometric rules and alter scaling equations at different organization scales remains to be elucidated. Finally, epigenetic marks along the genome can repress the expression of a large number of genes. For instance, methylated regions, which have reduced RNA transcription, represent a significant portion of the genome (Seymour et al. 2014; Seymour and Becker 2017). Consistent with a link between genome methylation level and organism growth and size, the re-analysis of the amount of methylated regions measured on more than 1000 natural accessions of *A. thaliana* (Kawakatsu et al. 2016) with the growth-related traits measured on 409 accessions in common (Vasseur et al. 2018a) (but on different individuals cultivated in an independent experiment), revealed a negative correlation between individual growth rate and the fraction of methylated genome (*r* =  − 0.044, slope =  − 0.02, *P* = 0.002; unpublished data), which suggests that larger expressed genomes are associated with bigger plants and higher absolute growth rate. The potential allometric linkage between expressed genome size and growth rate could also rely on stoichiometric considerations. Indeed, individuals with large genomes are expected to produce more RNA (Kozłowski et al. 2003b), which is phosphorus consuming and could explain the positive relationship between growth rate and RNA/protein as well as phosphorus/protein ratios observed empirically (Elser et al. 2003). Consistently, a strong relationship has been observed between the number of ribosomal genes per cell and cell size across a large diversity of prokaryote and eukaryote organisms (Gonzalez-de-Salceda and Garcia-Pichel 2021). Collectively, these findings suggest that the number of expressed genes in the genome could be the key piece to link allometric relationships at different levels of phenotypic integration, as well as to reconcile allometry with other disciplines such as biological stoichiometry, quantitative genetics and physiology.

## Conclusion

How multiple traits at different organizational levels are phenotypically integrated to ensure the proper development of complex organisms in a coordinated way is a key question for modern biology. We advocate here that allometric relationships offer a promising avenue to tackle this issue because (i) they rely on well-documented empirical observations, (ii) their mechanistic bases have been studied at different scales, and (iii) they propose a mathematical toolkit to infer hypotheses and make predictions. We argue that future directions for allometry research should focus in priority on the genetic determinism of scaling variation, taking advantage of the development of next-generation sequencing technologies, as well as on how the expressed genome size scales with higher orders of phenotypic integration, from cell to whole organism and metabolism. We hope that this article will encourage novel experiments and interdisciplinary investigations to address allometric questions at different levels of phenotypic integration.
